# Mapping out overlapping connectivity patterns

**DOI:** 10.7554/eLife.106507

**Published:** 2025-03-20

**Authors:** Myrthe Faber, Koen V Haak

**Affiliations:** 1 Department of Cognitive Science and Artificial Intelligence, Tilburg School of Humanities and Digital Sciences, Tilburg University Tilburg Netherlands; 2 Donders Centre for Cognitive Neuroimaging, Donders Institute for Brain Cognition and Behaviour, Radboud University Nijmegen Netherlands

**Keywords:** hippocampus, memory, functional connectivity, dopamine, aging, Human

## Abstract

Untangling the functional organisation of a brain region crucial for memory and learning helps reveal how individual differences are linked to variations in recall ability, aging and dopamine receptor distribution.

**Related research article** Nordin K, Pedersen R, Falahati F, Johansson J, Grill F, Andersson M, Korkki SM, Bäckman L, Zalesky A, Rieckmann A, Nyberg L, Salami A. 2024. Two long-axis dimensions of hippocampal-cortical integration support memory function across the adult lifespan. *eLife* **13**:RP97658. doi: 10.7554/eLife.97658.

What can the brain configuration of a person tell us about their behaviour, cognitive abilities, and susceptibility to disease? Answering this question requires a deeper understanding of the functional organisation of the brain: how its various parts perform specific roles yet manage to work together as a whole.

Traditionally, the brain has been mapped in terms of distinct areas based on structural information such as the kind of cells they feature. As such, boundaries between regions are often defined by abrupt changes in cell types. However, a more fine-grained organisation exists within brain areas, with certain neurons being more responsive to a particular task or stimulus than others. This functional heterogeneity emerges due to how the cells are wired, as their role is at least partly defined by their connections with the rest of the brain (Passingham et al., 2002). Unlike structural changes, connectivity often varies gradually across populations of cells, with transitions being smooth or abrupt depending on whether they take place within or between networks.

Now imagine that you want to create a detailed spatial map of these connections to study individual differences in functional brain organisation and their impact on cognition, behaviour and susceptibility to disease. A key challenge will be that multiple connectivity patterns may coexist and overlap, especially in zones where many brain networks converge (Jbabdi et al., 2013). This is called functional multiplicity. Without first disentangling these patterns and labelling each of them separately, it is impossible to obtain maps that can be used to examine individual variations and their impact on cognition (Haak and Beckmann, 2020). Now, in eLife, Kristin Nordin (Karolinska Institute, Stockholm University and Umeå University, all in Sweden) and colleagues report having carefully teased apart the various modes of functional organisation within the hippocampus, and how these relate to individual variations in memory, dopamine receptor distribution and aging (Nordin et al., 2024).

The human hippocampus is a deep brain structure essential for learning, spatial navigation and a type of memory that allows us to remember what event took place at a certain date and location. The researchers – who are based at institutes in Sweden, the Netherlands, Germany and Australia – noted that previous studies into the functional organisation of this region often had inconsistencies and hypothesised that these could be the result of functional multiplicity (Genon et al., 2021). To test this, they applied connectopic mapping on brain data obtained via functional MRI (fMRI). This method, which includes an advanced type of data processing known as non-linear manifold learning, can map out continuous changes in connectivity between cell populations, revealing the spatial organisation of connectivity patterns (Haak et al., 2018).

The analyses revealed at least two overlapping patterns of hippocampal connectivity, both stretching across the long axis of the structure (Figure 1). The first (called G1) showed that cells in the posterior part of the hippocampus tended to connect to ‘input areas’ that form representations, while those in the anterior part linked with regions that modulate these representations. The second mode of organisation (G2) captured the fact that the anterior and posterior ends of the hippocampus connect to ‘unimodal’ areas handling a single type of information, while the middle section links to ‘transmodal’ regions that integrate multiple types of inputs. This overall ‘unimodal-to-transmodal organisation’ has previously been linked to dopamine receptor distribution and signalling (Pedersen et al., 2024).

**Figure 1.**
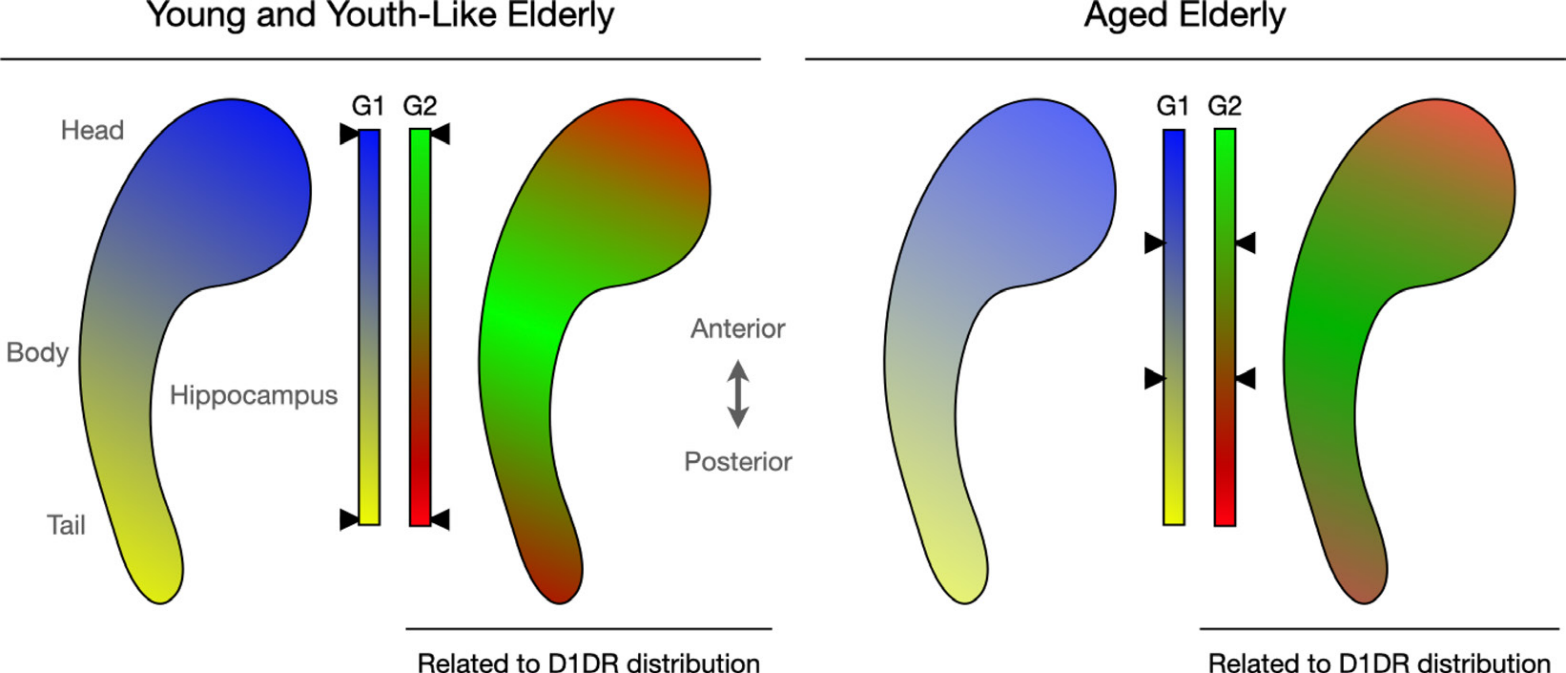
Connectopic mapping helps capture the functional organisation of the hippocampus. Nordin et al. investigated connectivity patterns in the human hippocampus (bean-like shape), a region important for memory and other cognitive tasks. The methods they applied allowed them to create spatial maps in which groups of cells with similar connectivity receive a similar colour. Smooth transitions in connectivity, for instance within a network, are then reflected by similar colours, whereas abrupt transitions, for instance between networks, are indicated by sharper colour changes. Two main patterns of organisation emerged (G1 and G2), each featuring two types of cell networks (in blue and yellow for G1, red and green for G2) linking to distinct brain areas. Further analyses examined individual variations in these patterns, in particular how they changed with age. This showed that variations in the spatial layout of G2 correlated with individual differences in the distribution of D1DR, a dopamine receptor crucial for hippocampal function. In addition, the analyses revealed that some older adults displayed ‘youth-like’ modes of organisation, with spatial patterns resembling those observed in young people (left). Those with ‘aged’ modes (right), on the other hand, had connectivity patterns indicative of decreased functional differentiation; demarcations between various connectivity networks became blurred (as represented by the black arrows next to the colour bars pointing to more blended colours in older adults compared to their young or ‘youth-like’ counterparts).

A third connectivity pattern (G3) also emerged, this time across the short axis of the hippocampus. It appeared to map onto well-known structural sub-regions, but Nordin et al. are appropriately cautious with their interpretation because increasingly higher order patterns are also increasingly subtle – and therefore increasingly difficult to map reliably.

Next, the team examined how hippocampal connectivity differed between individuals. They applied spatial statistical models to the connectopic mapping results. These capture how much of an area is functionally connected to another, and how smooth or abrupt connectivity transitions are (Haak et al., 2018). This approach returned three key observations.

First, in line with previous work, individual differences in the G1 mode of organisation were found to be predictive of memory performance in younger but not older adults (Przeździk et al., 2019), while G2 predicted memory performance across the adult lifespan. Second, older individuals with a more ‘youth-like’ connectivity profile had better memory performance. Analyses of their G1 patterns showed sharper demarcations between networks connecting to the input and input modulation areas, while G2 patterns showed sharper demarcations between the networks connecting to the unimodal and to transmodal areas, as in younger people. Third, G2 covaried with individual differences in dopamine receptor distribution, suggesting that this mode constitutes a connectivity pattern associated with dopamine signalling, just as the brain’s overall unimodal-to-transmodal organisation. The ‘blurring’ of G2 in older people might therefore be related to decreased dopamine availability.

These results will help understand how the hippocampus supports memory, learning and spatial navigation, and why these abilities decline with age or disease. In particular, the finding that dopamine receptor distribution covaries with specific hippocampal modes of organisation will likely have a major impact. Dopamine plays a crucial role in the hippocampus, and abnormal dopamine signalling in this brain region has been linked to disorders like Alzheimer’s and Parkinson’s disease (Edelmann and Lessmann, 2018). The work by Nordin et al., as well as similar studies in the striatum and other brain structures relying heavily on dopamine (Oldehinkel et al., 2022), suggests that it is possible to study dopamine signalling using fMRI data rather than invasive molecular neuroimaging methods. Ultimately, this may make it possible to identify new fMRI-based biomarkers of early Alzheimer’s and Parkinson’s disease.
